# Tracking Symptom Change Across Therapy Sessions in Virtual Reality Cognitive Behavioral Therapy Versus Cognitive Behavioral Therapy for Paranoid Ideation: A Secondary Analysis of a Randomized Controlled Trial

**DOI:** 10.1093/schbul/sbag068

**Published:** 2026-05-09

**Authors:** Maureen Berkhof, Elisabeth Christine Dorothée Van Der Stouwe, Wim Veling, Chris Neeltje Wil Geraets

**Affiliations:** Department of Psychiatry, University of Groningen, University Medical Center Groningen, 9700 RB Groningen, The Netherlands; Department of Psychiatry, University of Groningen, University Medical Center Groningen, 9700 RB Groningen, The Netherlands; Department of Psychiatry, University of Groningen, University Medical Center Groningen, 9700 RB Groningen, The Netherlands; Department of Psychiatry, University of Groningen, University Medical Center Groningen, 9700 RB Groningen, The Netherlands

**Keywords:** virtual reality, paranoia, psychosis, session, cognitive behavioral therapy, exposure

## Abstract

**Study Design:**

In this secondary analysis of a randomized controlled trial, individuals aged 18-65 with a psychotic disorder and paranoid ideation were recruited. Ninety-eight participants were randomized to maximally 16 sessions of VR-CBTp (*n* = 48) or CBTp (*n* = 50). Self-rated symptom severity, functional impairment, and clinician-rated illness severity were assessed at each therapy session. Analyses on change scores between the first and final session were performed to assess overall improvement, and multilevel regression models explored in-depth session differences.

**Study Results:**

Clinician-rated paranoia and avoidance improved more rapidly in VR-CBTp than CBTp, with differences emerging from session 3. Patients reported similar progress in both groups over time on symptoms and functional impairment. A greater proportion of VR-CBTp participants completed treatment early, requiring fewer sessions than the CBTp group.

**Conclusions:**

Therapists observed faster symptom recovery in paranoia and avoidance in VR-CBTp while requiring fewer sessions than standard CBTp. These findings suggest that VR-CBTp may be more efficient in duration, while broader effects on access and therapist workload remain to be determined.

## Introduction

Paranoid ideation is a common and distressing symptom in psychotic disorders, often associated with significant functional impairment and reduced quality of life.^1^^,^^2^ Cognitive behavioral therapy (CBTp) is the core psychological intervention recommended by international treatment guidelines for individuals with psychotic disorders.^3–8^ However, CBTp is significantly underutilized in clinical practice. A recent meta-analysis across high-income countries found that fewer than 30% of patients receive CBTp,^9^ often due to a shortage of trained therapists, limited time, and financial constraints.^10^

Reducing the duration of therapy without compromising effectiveness is therefore a key priority. International guidelines typically recommend at least 16 CBTp sessions.^11^ One meta-analysis suggested that 6-10 sessions can be effective,^12^ but an individual participant data meta-analysis, demonstrated better outcomes with longer treatments,^13^ consistent with a minimal dose of 15 and an optimal dose of 25 sessions reported by a study that assessed treatment response after 5, 15, 25, and 45 sessions.^14^ In contrast, another recent individual participant data meta-analysis found no influence of CBTp treatment duration on effectiveness.^15^

These findings raise the question how treatment efficiency of CBTp could be improved. Over the past decade, virtual reality (VR)-CBTp has emerged as a promising innovation to enhance treatment efficiency for paranoid ideation.^16–18^ Unlike standard CBTp, which typically starts with cognitive interventions during several sessions before introducing behavioral interventions, VR-CBTp allows for immediate engagement in immersive, personalized exposure exercises.^19^^,^^20^ These virtual environments can lower the threshold for behavioral experiments and facilitate the reduction of avoidance and safety behaviors—key maintaining factors in paranoia.^21^^,^^22^ As these key maintaining factors can be targeted faster in a personalized way in VR-CBTp, this may accelerate change, give stronger effects over sessions and reduce the number of sessions needed to achieve clinical improvement. Prior research has shown that reductions in safety behavior, rather than cognitive biases, are more strongly associated with decreases in paranoid ideation.^23^^,^^24^ Because these behavioral changes unfold gradually, session-by-session analysis can clarify when therapeutic effects emerge.

VR-CBTp has demonstrated large effect sizes compared to treatment as usual in 2 trials: one using a 16-session protocol^17^ and another using a single-session intervention.^18^ In the recent TOPIC-VR randomized clinical trial (RCT), the first head-to-head comparison of 16 sessions VR-CBTp and CBTp, we found that both treatments showed improvement in paranoid ideation over time, with VR-CBTp showing somewhat greater improvement.^16^ Another recent large RCT found similar results comparing 10 sessions of CBTp with VR-CBTp.^25^

To our knowledge, the VR-CBTp trials have reported solely on pre-, post-, and follow-up treatment outcomes.^17^^,^^18^^,^^25^ Analyzing recovery trajectories across sessions offers insight into the pace and timing of therapeutic change and can inform about potential treatment efficiency gains. Because VR-CBTp allows early introduction of immersive, therapist-controlled exposure, directly targeting proximal maintaining mechanisms such as safety behaviors, stronger within-treatment improvement can be expected, even in trials where posttreatment outcomes converge.

Session-by-session analyses for CBTp comparing 2 treatments are rare, and we only found one recent trial comparing the Feeling Safe cognitive therapy with befriending. They identified distinct trajectories for persecutory delusions, and showed variability both between the interventions and within, with subgroups following different patterns of change from session to session.^26^^,^^27^ Session-by-session analyses have also been applied in other areas of psychotherapy research, particularly for identifying different symptom trajectories in CBT for depression, anxiety, and psychosis.^28–30^

In this secondary analysis of TOPIC-VR RCT data,^16^ we examined symptom change across individual therapy sessions. Including both clinician and patient perspectives is essential: clinicians observe behavioral progress, while patients report subjective experiences that influence motivation and engagement—key factors in treatment adherence and outcomes.^31^ Therefore, using repeated clinician- and self-rated measures of paranoia, avoidance, and functioning, we aimed to characterize the course of symptom reduction and assess whether VR-CBTp leads to faster or more efficient improvement than CBTp. We hypothesized that VR-CBTp would result in quicker and stronger symptom reduction between sessions and requiring fewer sessions to achieve comparable outcomes compared to standard CBTp.

## Methods

### Study Design

This study is a secondary analysis of data from the multicenter TOPIC-VR RCT comparing 2 interventions: VR-CBTp and CBTp.^20^ The primary outcomes have been published elsewhere.^16^ The current study focused on the assessments that were completed during each treatment session. The study was approved by the medical ethical board of University Medical Centre Groningen, Groningen (NL66850.042.18), and conducted in accordance with the Declaration of Helsinki. The study has been registered in the Netherlands Trial Register (now CCMO register; trial number NL7758) and was funded by the Brain Foundation Netherlands (grant HA2017.01.04).

### Participants

Participants were patients with a psychotic disorder from 6 Dutch mental health institutions and 1 Belgian mental health institution. Inclusion criteria were: (1) DSM-5 diagnosis of schizophrenia spectrum or other psychotic disorder, as documented in the medical record; (2) at least a moderate level of paranoid ideation (Green Paranoid Thoughts Scale (GPTS)^32^ > 40); and (3) age 18-65 years. Exclusion criteria included: (1) an estimated IQ below 70; (2) insufficient command of Dutch language; and (3) receiving CBTp for paranoia within the past year.

### Interventions

VR-CBTp and CBTp both consisted of up to 16 individual on-site sessions, each lasting 60-75 min, conducted within an 8-12 week timeframe. During sessions the session measurements were completed. Interventions were delivered by therapists trained in both protocols. Therapists had at least a postgraduate CBTp qualification and minimally 6 months experience in psychosis treatment. They participated in monthly 2-h group supervision sessions for both interventions. Additionally, all individually tailored case conceptualizations, were reviewed and refined by supervisors after the second session. The largest difference between the protocols concerned the type and timing of exposure/behavioral exercises: in CBTp, in vivo exposure was introduced after session 7, whereas VR-CBTp allowed VR-based exposure from session 3 onward. Although exposure was introduced earlier in the VR-CBTp protocol, therapists were allowed to adapt the pacing of CBTp sessions, meaning exposure could occur earlier when clinically appropriate.

### CBTp

The Dutch CBTp protocol for paranoid ideation (Gedachten Uitpluizen) was followed.^33^ In the initial sessions (inventory phase), therapists and patients constructed individual case formulations, fostering an understanding of current paranoid ideas, feelings, and behavior with a “safety circle.” The safety circle is a personalized overview outlining paranoia-related triggers and associated safety behaviors, serving as a checklist to verify that the core maintaining factors of paranoia were sufficiently targeted. Then, the intervention phase started with cognitive interventions such as cognitive restructuring by means of Socratic dialogue, pie chart techniques, and probability calculations to challenge unhelpful thought patterns and threat expectations. After session 7, sessions emphasized behavioral interventions (eg, in vivo exposure and behavioral experiments) to experience safety despite high levels of anxiety and corresponding threat expectations. As part of the protocol, the safety circle was monitored throughout treatment and reviewed to determine readiness for termination.

### VR-CBTp

The VR-CBTp protocol was adapted from the standard CBTp protocol with VR exercises. In the first 2 session participants tried the VR to get used to it. Starting from session 3 onwards, 40 min of each session were dedicated to immersive VR exercises. This 40-min period represented the intended duration per protocol. Patients wore an Oculus Rift head-mounted display and navigated virtual environments using a controller. Therapists operated avatars in real time—modulating facial expressions, gestures, and speech via a microphone with voice distortion—to engage patients in interactive role-plays.

The VR software, *Social Worlds* (developed by CleVR BV), offered a range of animated environments such as a café, shopping street, supermarket, bus, office, park, and living room. Personalization was a key feature: therapists tailored the environments, selected the number, gender, and appearance of avatars, and adjusted their behavior (eg, hostility or ambiguity) to match each patient’s therapeutic needs and goals. These settings were designed to elicit paranoid ideation and distress. During sessions, therapists guided patients to drop safety behaviors, test paranoid beliefs, and practice new behaviors within these virtual scenarios.

### Early Completion of Treatment

Treatment could be completed early when (1) exposure exercises and behavioral experiments (conducted either in vivo or VR) no longer elicited anxiety or distress, and (2) predefined treatment goals behaviors had been consistently achieved. Because exposure exercises occurred in VR for VR CBTp and in real life situations for CBTp, the criteria for determining when exposure no longer evoked distress were necessarily modality-specific. In cases where patients had achieved their target behaviors in daily life without having completed in vivo exposure sessions during CBTp, treatment could also be completed without requiring Subjective Units of Distress (SUD) scores. Before concluding treatment, all elements of the individualized safety circle were carefully reviewed to ensure that triggers of paranoid ideations had been addressed. A SUD score of 0 on established paranoia cues across 2 consecutive sessions was required (for VR-CBTp this concerned cues in VR, and for CBTp in real life), alongside interim assessments indicating sustained reductions in paranoia, distress, and avoidance. Subsequently, a supervisory consultation was conducted to review the case, and if mutual agreement was reached between therapist and supervisor, the final session was planned. Supervisors were not blind to treatment allocation.

### Procedures

Potentially eligible patients were asked by their clinicians if they were interested in participating. Then researchers provided verbal and written study information and conducted eligibility screening. If patients decided to participate, written informed consent was obtained, and followed by completing the GPTS. If the GPTS score was >40, patients were eligible, and the baseline assessment continued. Randomization was done after baseline, using block randomization with a block size of 8 assignments per mental health center. At the start of each session, participants completed the Sheehan Disability Scale (SDS)^34^ and Visual Analog Scale (VAS) items assessing symptom severity. At the end of each session, therapists completed the Clinical Global Impression (CGI).^35^ These were completed in the session workbooks.

### Outcome Measures

#### Clinical Global Impression (CGI)

The CGI is a clinician-rated measure consisting of 3 items: the global improvement compared to the start of treatment, global severity of paranoia ideation since the previous session, and global severity of social avoidance since the previous session.^35^^,^^36^ Items were rated on a 7-point scale.

#### Functional Impairment (SDS)

The SDS is a self-report questionnaire that assesses functional impairment (in the past week) caused by unusual experiences—in this case, paranoid ideation—across 3 key life domains: work/school, social, and family life/home.^34^ Each domain is rated on a 10-point scale, ranging from 0 (“not at all impaired”) to 10 (“extremely impaired”). Two items that record missed and unproductive work/school days were excluded because they were often structurally inapplicable and showed inconsistent completion (eg, participants without work/school obligations completing them, and those with obligations leaving them blank), resulting in high and non-random missingness (17% and 18%) and reduced validity.

#### Symptom Severity (VAS)

The VAS comprised 9 items rated on a VAS ranging from 0 to 100. Items were rated about the past week. Seven items were derived from paranoia related items from the experience sampling diary methodology (ESM)^17^^,^^37^ and 2 items were based on the safety behavior questionnaire (SBQ)^38^ concerning avoidance. For the 7 ESM VAS items a principal component analysis (PCA) was performed. The PCA with oblique rotation and Kaiser normalization for the person-centered data identified 2 factors according to the Kaiser criterion of an eigenvalue >1. The paranoia subscale consisted of 5 items with factor loadings ranging from 0.72 to 0.82: “I feel that others might hurt me,” “I feel that others dislike me,” “In company, I felt unsafe,” “I felt anxious,” and “I feel suspicious.” The social acceptance subscale included 2 items—“I felt accepted in company” (listed twice, possibly in error)—with high factor loadings of 0.90 and 0.88. Subscale scores were calculated by averaging the respective items. In addition, the 2 SBQ-derived avoidance items were analyzed separately: “I have avoided situations/activities to prevent danger or fear” and “I have endured situations/activities that I could not avoid, with difficulty.”

### Statistical Methods

IBM SPSS Statistics version 29.0.1.0 was utilized for all analyses. Both intention-to-treat and per protocol analyses were performed, as a form of sensitivity testing. The intention-to-treat analysis served as the primary analytic approach, in line with the original TOPIC-VR trial, and the per protocol analysis was conducted as a sensitivity analysis to assess the robustness of the findings. A *P*-value of <.05 was considered statistically significant. No correction for multiple testing was applied because this was an exploratory secondary analysis addressing a single overarching research question. Strict correction would increase Type II error and obscure clinically relevant patterns; results should therefore be interpreted with caution.

Group differences in treatment dropouts, successful early completers, number of sessions completed, and global improvement were analyzed with chi-square, *t*-test or Mann–Whitney U test.

To evaluate treatment effects, both change score comparisons and multilevel regression analyses were employed. Change scores offered a straightforward summary of overall improvement and facilitated clinically meaningful interpretations. Multilevel models complemented this by using all available data, accounting for individual variability, and capturing change trajectories over time.

(1) Change score analyses were done, comparing the difference between the final and first session using independent t-tests or non-parametric Mann–Whitney U tests. Change scores were appropriate as baseline levels were comparable between groups due to randomization.^39^ If session 1 data were missing, values from session 2 or 3 were imputed. Additionally, the Last Observation Carried Forward (LOCF) method was used to impute values up to session 16 for all participants, including dropouts and early completers. This approach supported both change score analyses and the generation of Reliable Change Index (RCI) graphs.
(2) Multilevel regression analyses were conducted on all available data (without any imputations) for the CGI, VAS, and SDS. Models included fixed effects for group, session number, and their interaction (group × session number), as well as a random intercept for participant and a random slope for session number. Restricted maximum likelihood estimation and an unstructured covariance matrix were applied.

In case a significant session-by-group interaction was found, we further explored the speed of change by using the 95%-RCI.^40^ The RCIs were calculated based on the SD and reliability coefficient. As a reliability coefficient, the test–retest variance between session 1 and session 2 scores was used (since this can be calculated for single items as well). The percentage of participants exceeding the RCI per session and group was calculated and visualized using bar graphs.

## Results

Between October 1, 2019, and July 31, 2023, 98 participants were randomized to VR-CBTp (*n* = 48) or CBTp (*n* = 50). Baseline characteristics were comparable across groups in terms of age, gender, education, and diagnoses (see Supplement 1). The mean age was 35.5 years (SD = 12.9) in the VR-CBTp group and 36.1 years (SD = 12.3) in the CBTp group, and most were male (68.8% in VR-CBTp; 78.0% in CBTp). The most common diagnoses were Unspecified Schizophrenia Spectrum and Other Psychotic Disorder, and Schizophrenia. Antipsychotic medication was prescribed to 89.6% of participants in the VR-CBTp group and 78.0% in the CBTp group.

### Treatment Dropouts, Early Completers, and Duration

**Figure 1:**
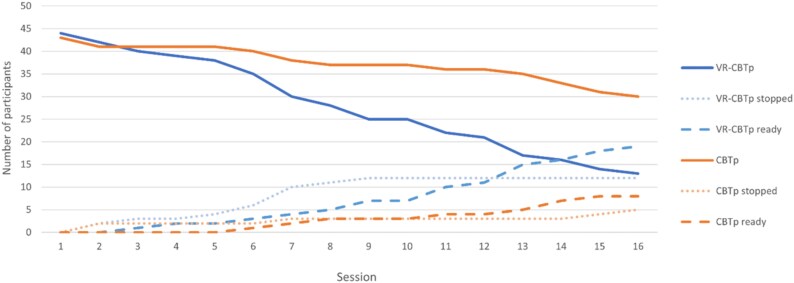
Session Plot per Group Showing the Number of Dropouts, Completers, and Number Attending Each Session (*n* = 44 in Each Group).

Figure 1 shows the distribution of dropouts, treatment completers, and number attending each session across both groups. The number of participants completing treatment increased more rapidly in the VR-CBTp group, although the dropout rate was also higher. The dropout rate was 37.5% (*n* = 18) for VR-CBTp and 24.0% (*n* = 12) for CBTp, a difference that was not statistically significant (χ^2^ = 2.1, *P* = .15). Of those who dropped out, 10 participants never started treatment (VR-CBTp: *n* = 4, CBTp: *n* = 6). Four participants discontinued due to treatment-related reasons, with 3 in the VR-CBTp group and 1 in the CBTp group reporting that the intervention was too intensive (Table 1).

**Table 1:** Treatment Characteristics

	**VR-CBTp** ***n* = 48**		**CBTp** ***n* = 50**	
**Number of sessions (intention-to-treat)**	**9.4**	**(5.6)**	**12.3**	**(6.0)**
**Treatment completers** (per protocol)	***n* = 30**	**(62.5%)**	***n* = 38**	**(76.0%)**
Number of sessions mean	12.7	(3.9)	15.1	(2.5)
Number of sessions				
2 sessions	1	(3.3%)	0	
5 sessions	1	(3.3%)	1	(2.6%)
6 sessions	1	(3.3%)	1	(2.6%)
7 sessions	1	(3.3%)	0	
8 sessions	1	(3.3%)	0	
10 sessions	3	(10.0%)	0	
11 sessions	1	(3.3%)	0	
12 sessions	3	(10.0%)	1	(2.6%)
13 sessions	2	(6.7%)	2	(5.3%)
14 sessions	2	(6.7%)	1	(2.6%)
15 sessions	1	(3.3%)	2	(5.3%)
16 sessions	13	(43.3%)	30	(78.9%)
**Treatment drop-outs**	***n* = 18**	**(37.5%)**	***n* = 12**	**(24.0%)**
Number of sessions				
0 sessions	4	(22.2%)	6	(50.0%)
1 sessions	2	(11.1%)	2	(16.7%)
2 sessions	1	(5.5%)	0	
3 sessions	1	(5.5%)	0	
4 sessions	1	(5.5%)	0	
5 sessions	2	(11.1%)	1	(8.3%)
6 sessions	4	(22.2%)	0	
7 sessions	1	(5.5%)	0	
8 sessions	2	(11.1%)	0	
10 sessions	0		1	(8.3%)
12 sessions	0		1	(8.3%)
14 sessions	0		1	(8.3%)
**Reasons for dropout**				
Symptom reduction prior to start	2		0	
Exacerbated symptoms/no trust in care	1		4	
Other treatment required	4		3	
Unwilling/lost contact/unknown	6		2	
Treatment too intensive	3		1	
Assessment too intensive	1		1	
Severe physical illness/imprisonment	1		1	

Data are *n* (%) or mean (SD).

Among participants who completed treatment, a significantly higher proportion in the VR-CBTp group (57%, *n* = 17) were classified as early completers compared to CBTp (21%, *n* = 8) (χ^2^ = 9.1, *P* = .002). The average number of sessions received by treatment completers was significantly lower in the VR-CBTp group (*M* = 12.7, SD = 3.9) than in the CBTp group (*M* = 15.1, SD = 2.5), with a mean difference of 2.5 sessions (U = 738.5, *P* < .001).

Of the 88 participants who started treatment, session workbooks were missing for 5 (VR-CBTp: *n* = 2, CBTp: *n* = 3). The remaining 83 participants attended a total of 1000 sessions. Data were available for over 90% of these sessions for all measures (VAS: *n* = 973, SDS: *n* = 942, CGI: *n* = 915).

### Clinician-Rated Global Improvement

Table 2 presents clinician-rated global improvement at the final session compared to the start. In the intention-to-treat analysis, 64% of the CBTp group were rated as “much” or “very much” improved, compared to 55% in the VR-CBTp group. Among treatment completers (per protocol), this pattern reversed: 75% of participants in the VR-CBTp group were rated as “much” or “very much” improved, compared to 67% in the CBTp group. However, these differences were not statistically significant according to chi-square tests. Similarly, multilevel regression analyses on all available session data showed a significant main effect of time but not of group or the interaction of group-by-time (see Table 3 for the intention-to-treat results, and Supplement 2 for per protocol results).

**Table 2:** Global Improvement as Therapists Rated at the Final Session (*n* = 79, Intention-to-Treat, Minimal 2 Sessions Needed)

	**VR-CBTp**	**CBTp**	**VR-CBTp**	**CBTp**
	**Intention-to-treat**		**Per protocol**	
	***N* = 40**	***N* = 39**	***N* = 28**	***N* = 36**
1 Very much improved (%)	20.0	15.4	25.0	16.7
2 Much improved (%)	35.0	48.7	50.0	50.0
3 Minimally improved (%)	25.0	25.6	21.4	25.0
4 No change (%)	15.0	7.7	3.6	5.6
5 Minimally worse (%)	2.5	2.6		2.8
6 Much worse (%)	2.5			
7 Very much worse (%)				

*n* = 79 as 4 out of 83 participants dropped out after session 1 and thus had no global improvement rating. In case the final session rating was missing, the last measurement was imputed.

**Table 3:** Intention-to-Treat Treatment Outcomes During Sessions and Differences Between VR-CBTp and CBTp

		**Main effect group**		**Main effect session**		**Interaction**	
		***b* (95% CI)**	***P***	***b* (95% CI)**	***P***	***b* (95% CI)**	***P***
**Clinician-rated**							
Global Improvement	CGI	−0.03 (−0.32 to 0.25)	.81	−0.14 (−0.17 to −0.12)	**<.001**	−0.02 (−0.07 to 0.02)	.35
Paranoia	CGI	0.02 (−0.52 to 0.57)	.93	−0.14 (−0.18 to −0.09)	**<.001**	−0.08 (−0.14 to −0.02)	**.02**
Avoidance	CGI	0.12 (−0.46 to 0.70)	.27	−0.14 (−0.18 to −0.10)	**<.001**	−0.08 (−0.14 to −0.02)	**.01**
**Self-rated**							
Paranoia	VAS	2.4 (−8.5 to 13.4)	.66	−1.1 (−1.7 to −0.4)	**<.001**	−0.3 (1.2 to 0.6)	.50
Social acceptance	VAS	−9.9 (−18.9 to −1.0)	**.03**	0.5 (−0.0 to 1.0)	**<.001**	0.4 (−0.4 to 1.3)	.30
Avoidance	VAS	−4.0 (−16.2 to 8.2)	.52	−1.4 (−2.0 to −0.7)	**<.001**	0.2 (−0.8 to 1.1)	.73
Enduring threat situations	VAS	1.0 (−11.1 to 13.1)	.87	−1.1 (−1.7 to −0.5)	**<.001**	−0.4 (−1.3 to 0.5)	.41
School/work	SDS	0.26 (1.17 to 1.70)	.72	−0.21 (−0.29 to −0.13)	**<.001**	0.03 (−0.10 to 0.15)	.65
Social life	SDS	0.43 (−0.68 to 1.54)	.44	−0.16 (−0.22 to −0.09)	**<.001**	−0.07 (−0.17 to 0.03)	.17
Family life/home	SDS	0.09 (−0.98 to 1.17)	.86	−0.12 (−0.17 to −0.06)	**<.001**	−0.04 (−0.12 to 0.05)	.40

### Clinician-Rated Paranoia and Avoidance (CGI)

Change scores between the final and first session showed no significant difference between the groups in severity of paranoia (*t*(75) = −0.7, *P* = .50) or avoidance (*t*(75) = 0.1, *P* = .93). The mean change in paranoia was −1.9 (SD = 1.7) for VR-CBTp and −1.7 (SD = 1.6) for CBTp. For avoidance, both groups showed an identical mean change of −1.9, with SD of 1.7 (VR-CBTp) and 1.3 (CBTp). Per protocol analyses yielded similar results, with no significant group differences. The mean change in paranoia was −2.2 (SD = 1.6) for VR-CBTp and −1.8 (SD = 1.4) for CBTp. For avoidance, the mean change scores were −2.1 (SD = 1.8) for VR-CBTp and −1.9 (SD = 1.4) for CBTp.

Multilevel regression models confirmed that overall, both treatments reduced paranoia and avoidance. Importantly, significant group-by-session interaction effects were observed for both outcomes, indicating a faster rate of symptom reduction in the VR-CBTp group (Table 2). In per protocol analyses, the interaction effect remained significant for avoidance (*P* = .04), but not paranoia (*P* = .06).

**Figure 2:**
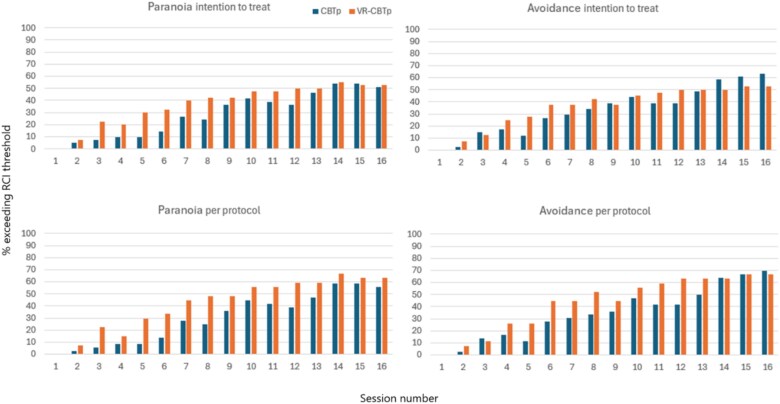
Bars Indicate the Percentage of Participants Exceeding the RCI Threshold for CGI Paranoia and Avoidance, Both intention-to-treat and per protocol. Data were imputed using last observation carried forward. Intention-to-treat: VR-CBTp *n* = 40, CBTp *n* = 41. Per-protocol: VR-CBTp *n* = 27, CBTp *n* = 36. Lower *n* values were due to missing workbooks or fewer than 2 sessions.

To further assess the speed of change in CGI paranoia and avoidance, we calculated the 95% RCI, which indicated that a reduction of 2 points represented a reliable improvement for both items. As shown in Figure 2, a higher percentage of participants in the VR-CBTp group surpassed the RCI threshold earlier in treatment compared to the CBTp group, particularly for paranoia, with divergence beginning around session 3.

### Self-Rated Symptoms (VAS)

On average, participants in both groups reported reductions in paranoia, avoidance, and enduring threat situations between the first and final session. Specifically, paranoia decreased by −12.5 points (SD = 21.8) in the VR-CBTp group and −17.3 (SD = 27.2) in the CBTp group (U = 868.5, *P* = .39). Avoidance scores declined by −13.4 points (SD = 32.7) in the VR-CBTp group and −17.6 points (SD = 32.7) in the CBTp group (U = 816.0, *P* = .72). Enduring threat situations improved by −12.8 points (SD = 26.0) in the VR-CBTp group and −16.1 points (SD = 31.5) in the CBTp group (*t*(77) = 0.5, *P* = .62). None of these differences between groups were statistically significant. Regarding social acceptance the VR-CBTp group improved 8.4 points (SD = 29.3) compared to 6.7 for CBTp (SD = 20.2), which was also not significant (*t*(77) = 0.3, *P* = .76).

Per protocol analyses yielded similar findings, however, the mean change scores were much more similar for paranoia (M_VR-CBT_ = −17.1, SD = 20.3; M_CBT_ = −17.6, SD = 27.6) avoidance (M_VR-CBT_ = −15.4, SD = 35.2; M_CBT_ = −16.5, SD = 30.6), and enduring threat situations (M_VR-CBT_ = −16.5, SD = 24.0; M_CBT_ = −15.8, SD = 31.8). For social acceptance, differences were larger; VR-CBTp improved 11.0 points (SD = 26.2) compared to 5.2 for CBTp (SD = 19.7).

The multilevel regression analyses also partly reflected the difference in social acceptance, by showing a significant main effect of group. Furthermore, for paranoia, avoidance, and enduring threat situations, only significant effects of session were found and no effect of group or interactions.

### Self-Rated Functioning (SDS)

Analyses of SDS change scores revealed a significant group difference in the Work/School domain favoring CBTp (U = 723, *P* = .04). Participants in the CBTp group reported greater improvement (M = −3.3, SD = 3.7) compared to VR-CBTp (M = −1.6, SD = 3.3). No significant group differences were found for the Social Life domain (M_VR-CBT_ = −2.6, SD = 2.8, M_CBTp_ = −2.3, SD = 2.9, *t*(75) = −0.5, *P* = .59) or Family Life (M_VR-CBT_ = −2.4, SD = 3.0; M_CBTp_ = −1.2, SD = 3.1, t(75) = −1.7, *P* = .09). Per protocol analyses showed similar results. Additionally, multilevel regression analyses showed a consistent main effect of session across all domains, indicating improvement over time. However, no significant main effect of group or group-by-session interaction was found.

## Discussion

This is the first study to examine differences between VR-CBTp and at the session level, investigating whether VR-CBTp leads to faster and stronger symptom reduction than standard CBTp in individuals with paranoid ideations. Contrary to our hypothesis, both treatments showed similar improvements in paranoia, avoidance, and global functioning, thus VR-CBTp did not result in stronger symptom reductions. VR-CBTp was associated with a more rapid decline in clinician-rated paranoia and avoidance symptoms over the course of treatment. Additionally, participants in the VR-CBTp group completed treatment in significantly fewer sessions and showed higher rates of early completion, suggesting greater efficiency.

Findings suggest that VR-CBTp may provide a more time-efficient treatment format, particularly in accelerating behavioral change, without compromising clinical benefit. This faster within group change likely stems from earlier use of behavioral strategies, such as exposure and behavioral experiments, in VR-CBTp. While we cannot definitively attribute the faster symptom reduction to these elements, they represent the most notable difference between the VR-CBTp and standard CBTp treatment protocols. The ability to tailor and repeat realistic exercises in safe virtual environments may accelerate therapeutic processes such as emotional processing, mastery, and self-efficacy. These mechanisms are central to the inhibitory learning model of exposure therapy, which posits that repeated confrontation with feared stimuli fosters new, non-threatening associations.^22^^,^^24^^,^^41^ Our results support this model and underscore the importance of behavioral strategies in treating paranoia.

Notably, the session-by-session analysis revealed that differences in clinician-rated paranoia and avoidance began to emerge around session 3, aligning with the start of VR exposure exercises. However, not all participants showed improvement—some demonstrated minimal or no change, as reflected in the RCI graphs. This highlights the need for further research into individual response patterns and potential moderators of treatment efficacy. For CBTp, improvement became more apparent after session 7, corresponding to the later introduction of in-vivo exposure in the standard protocol. This suggests that the timing of exposure may play a crucial role in the earlier changes observed in VR-CBTp. In standard CBTp, early exposure is often not feasible because patients typically require several sessions of cognitive preparation before confronting feared situations but the VR context allows for quick exposure.^42–44^ Prior work suggests that VR can make early exposure more feasible and acceptable because patients know that the environments are not real, lowering initial fear and increases willingness to engage.^45^ Furthermore, it removes logistical barriers to arranging in vivo exposure,^46^ and gives therapists greater control over the environment decreasing therapists worry about patient distress and dropout.^47^ Moreover, therapist-related factors can affect exposure implementation,^48^^,^^49^ yet these influences were likely limited in our trial given that all therapists were trained and provided both treatments.

Whereas we focused on difference in trajectories between treatments, Jenner and colleagues also examined variability within treatment, identifying distinct response patterns.^26^^,^^27^ One of the four response classes of the Feeling Safe intervention also showed fast recovery from session 3 similar to our study, the other 3 classes showed slower or no recovery. Future research could similarly explore such within-treatment trajectories in CBTp and VR-CBTp to inform more personalized intervention strategies.

Although clinician ratings showed greater decrease in paranoia and avoidance for VR-CBTp, these effects were not reflected in self-reported outcomes. This discrepancy may reflect differences in focus: clinicians ratings focused on behavior and symptom expression during sessions and impressions of the previous week, whereas VAS scales reflect momentary feelings and experience of participants.^38^^,^^39^ At the same time, patients may have experienced behavioral changes without being conscious of them. An additional potential explanation is that therapists may have had higher expectations for VR-CBTp, potentially introducing bias in their ratings and early-completion decisions. Interestingly, whereas VR-CBTp was associated with faster symptom reduction, CBTp showed greater improvement in self-rated work/school functioning. As we did not assess factors that might influence work or school functioning, this isolated difference is difficult to interpret and does not align with the otherwise similar pattern of self-rated outcomes across conditions.

Dropout was higher during VR-CBTp (37%) than CBTp (24%), with some participants in both groups dropping out before starting therapy. Most dropouts were unrelated to the interventions (eg, prioritizing other therapies, illness, or imprisonment). For VR-CBTp, practical issues such as traveling to VR locations and the strong early focus on exposure may have reduced tolerability for some.

Overall, intention-to-treat and per protocol analyses very showed similar patterns. With regard to clinician rated global improvement, intention-to-treat analyses suggested slightly higher (non-significant) improvement ratings for CBTp (64% rated as “much” or “very much improved”) compared to VR-CBTp (55%). In contrast, per protocol analyses showed the reverse pattern: 75% of VR-CBTp participants were rated as “much” or “very much improved” versus 67% in CBTp. This discrepancy may partly reflect the higher dropout among VR-CBTp participants due to practical challenges or early exposure demands, while individuals in CBTp who were less satisfied with progress might have still completed therapy, although we have no information about this in our data.

### Strengths and Limitations

This study is the first VR-CBTp study to systematically track symptom progression across individual sessions. Conducted in 7 mental health centers with regular clinical staff and consistent supervision, the study reflects real-world practice. The use of both clinician- and self-rated measures allowed for a comprehensive evaluation.

This study has several limitations. First, missing data from 5 participants and approximately 6% of session-level data may have reduced statistical power and precision. Second, the use of LOCF to impute missing values assumes symptom stability and may underestimate variability, particularly among early dropouts. Third, patients unwilling to travel due to severe avoidance may have been underrepresented, potentially limiting generalizability. Fourth, participants, therapists, and supervisors were not blinded to treatment condition, which may have introduced expectancy effects. The novelty of VR could have increased enthusiasm or engagement among both therapists and patients, potentially influencing outcome ratings and decisions about early treatment completion. Fifth, although both clinician- and self-rated measures were used, reliance on single-item CGI ratings and VAS scales may limit the depth and reliability of symptom assessment. Sixth, a further limitation is that exposure began earlier in VR-CBTp than in CBTp by protocol, while we did not record the actual amount or intensity of exposure. Therefore, we are unable to provide empirical evidence that differences in exposure account for the faster improvement. Also, we did not comprehensively assess other underlying mechanisms of change, such as improvements in safety behavior or self-efficacy. Although avoidance was included as a CGI item, reliance on a single-item rating provides only a limited proxy for these mechanisms. An additional key limitation for causal inference is the absence of a passive or inactive control condition, which precludes attributing improvements to either intervention. Finally, early termination criteria differed partly between conditions (VR cues for VR-CBTp vs. real-life cues for CBTp). This may have affected comparability of treatment completion and outcomes. Therefore, results should be interpreted with caution, and future research would benefit from defining termination criteria that apply equivalently across interventions.

### Implications and Future Research

Both VR-CBTp and CBTp showed similar within-treatment improvements in psychotic symptoms over time, but VR-CBTp was associated with greater time efficiency, requiring fewer sessions to achieve comparable outcomes. This has implications for mental health services, as VR-CBTp may reduce therapist workload and increase treatment capacity. Its flexibility makes it suitable for various settings, including delivery in clinics and potentially at home. This is particularly valuable for patients who avoid in vivo exposure, as VR offers a safe, immersive alternative. Although the initial investment in VR technology is higher, the potential for faster recovery and reduced therapist time suggests long-term cost-effectiveness. Formal cost-effectiveness analyses are therefore needed, including both direct (eg, equipment, therapist time) and indirect costs (eg, reduced healthcare use, improved functioning).

Further, future research should examine long-term outcomes of VR-CBTp and identify patient subgroups who benefit most (eg, through moderation or latent class trajectory modelling). Future trials should also evaluate whether VR-CBTp can be delivered effectively as a shorter therapy, for example by testing fixed reduced-session protocols against standard CBTp, while monitoring durability of effects and using passive control comparators. Additionally, studies should systematically record the duration of exposure to analyze whether the hypothesized mechanisms, such as enhanced exposure, correspond with the accelerated within-treatment changes in VR-CBTp.

Automated VR applications, potentially enhanced by generative AI, have the potential to make psychological therapy more accessible and scalable. They could be used to increase treatment intensity by complementing face-to-face sessions with home-based VR exercises or as booster sessions after treatment.^50^ As VR technology advances, future research should explore whether elements of VR-CBTp can be delivered in partially self-guided or partially automated formats, while ensuring clinical safety and appropriate therapeutic oversight.

## Conclusion

Both VR-CBTp and standard CBTp both resulted in substantial reductions in paranoia and avoidance scores over time. VR-CBTp was associated with faster clinician-rated symptom reduction and treatment completion in fewer sessions compared to standard CBTp. These quicker improvements, particularly in avoidance and paranoia, may reflect earlier behavioral practice facilitated by VR, though this hypothesis requires further investigation. Given the shortage of trained psychologists and time constraints, VR-CBTp could potentially help increase treatment accessibility by enabling more patients to be treated in fewer sessions.

## Supplementary Material

sbag068_Supplemental_File
