# Supplementary material for: Tracking Symptom Change Across Therapy Sessions in Virtual Reality Cognitive Behavioral Therapy Versus Cognitive Behavioral Therapy for Paranoid Ideation: A Secondary Analysis of a Randomized Controlled Trial
Source: Schizophr Bull. 2026 May 9;52(3):sbag068. doi: 10.1093/schbul/sbag068 (PMC13156502; doi:10.1093/schbul/sbag068)
Supplement: sbag068_Supplemental_File [file sbag068_supplemental_file.docx]

| **Supplement 1. Baseline characteristics** | |  |  |
| --- | --- | --- | --- |
|  | **VR-CBTp n=48** | **CBTp n=50** |  |
| Age, years | 35.5 (12.9) | 36.1 (12.3) |  |
| Male | 33 (68.8%) | 39 (78.0%) |  |
| Female | 15 (31.3%) | 11 (22.0%) |  |
| Education |  |  |  |
| None or primary | 4 (8.3%) | 2 (4.0%) |  |
| Secondary | 22 (46.1%) | 23 (46.0%) |  |
| Vocational | 14 (29.2%) | 18 (36.0%) |  |
| Higher | 8 (16.9%) | 7 (14.0%) |  |
| DSM-5 psychosis spectrum diagnosis |  |  |  |
| Unspecified Schizophrenia Spectrum and Other Psychotic Disorder | 18 (37.5%) | 23 (46.0%) |  |
| Schizophrenia | 14 (29.2%) | 7 (14.0%) |  |
| Schizoaffective disorder | 5 (10.4%) | 5 (10.0%) |  |
| Delusional disorder | 6 (12.5%) | 5 (10.0%) |  |
| Other Specified Schizophrenia Spectrum and Other Psychotic | 2 (4.2%) | 6 (12.0%) |  |
| Disorder |  |  |  |
| Brief psychotic disorder | 1 (2.1%) | 4 (8.0%) |  |
| Schizophreniform disorder | 1 (2.1%) | 0 |  |
| Schizotypal personality disorder | 1 (2.1%) | 0 |  |
| Prescribed an antipsychotic medication | 43 (89.6%) | 39 (78.0%) |  |
| Data are n (%) or mean (standard deviation). |  |  |  |

**Supplement 2.** Per protocol treatment outcomes during sessions and differences between VR-CBTp and CBTp

|  |  | **Main effect group** | | **Main effect session** | | **Interaction group x session** | |
| --- | --- | --- | --- | --- | --- | --- | --- |
|  |  | b (95% CI) | *p* | b (95% CI) | *p* | b (95% CI) | *p* |
| ***Clinician-rated*** | | | | | | | |
| Global Improvement | CGI | -0.07 (-0.38:0.24) | .65 | -0.14 (-0.17:-0.11) | **<.001** | -0.03 (-0.08:0.02) | .23 |
| Paranoia | CGI | -0.21 (-0.80:0.38) | .48 | -0.14 (-0.18: -0.01) | **<.001** | -0.06 (-0.12:0.00) | .06 |
| Avoidance | CGI | -0.02 (-0.67:0.63) | .96 | -0.14 (-0.17:-0.10) | **<.001** | -0.07 (-0.13:0.00) | **.04** |
| ***Self-rated*** | | | | | | | |
| Paranoia | VAS | 0.5 (-11.6:12.6) | .93 | -1.1 (-1.7:0.5) | **<.001** | -0.6 (-1.5:0.4) | .22 |
| Social acceptance | VAS | -7.2 (-16.3:1.9) | .12 | 0.4 (-0.1:0.9) | **.002** | 0.5 (-0.3:1.3) | .20 |
| Avoided to prevent danger | VAS | -6.2 (-20.4:8.0) | .39 | -1.3 (-1.9:-0.7 | **<.001** | 0.2 (-0.8:1.2) | .69 |
| Endured dangerous situations | VAS | 0.5 (-12.8:13.8) | .94 | -1.2 (-1.8:-0.6) | **<.001** | -0.5 (-1.5:0.5) | .33 |
| School/Work | SDS | -0.38 (-2.00:1.23) | .64 | -0.22 (-0.30:-0.14) | **<.001** | 0.06 (-0.07:0.18) | .36 |
| Social Life | SDS | 0.02 (-1.22:1.27) | .97 | -0.17 (-0.23:-0.10) | **<.001** | -0.05 (-0.16:0.05) | .34 |
| Family Life/Home | SDS | -0.10 (-1.33:1.12) | .87 | -0.12 (-0.18:-0.07 | **<.001** | -0.02 (-0.10:0.07) | .72 |
